# Detection of Early Warning Signs in Autism Spectrum Disorders: A Systematic Review

**DOI:** 10.3390/children8020164

**Published:** 2021-02-22

**Authors:** José María Salgado-Cacho, María del Pilar Moreno-Jiménez, Yolanda de Diego-Otero

**Affiliations:** 1 Institute of Biomedical Research of Málaga (IBIMA), 29010 Malaga, Spain; salgadocacho@gmail.com; 2 Hogar Abierto, 29001 Malaga, Spain; 3 Faculty of Psychology, University of Málaga, 29010 Malaga, Spain; mpilar@uma.es; 4 Research Group PREVENT-Rare (PAIDI CTS456), 29009 Malaga, Spain; 5 International Institute of Innovation and attention to neurodevelopment and language I3NEL, 29011 Malaga, Spain

**Keywords:** ASD, early detection, risk markers, screening, theoretical study

## Abstract

Due to the exponential increase of autism spectrum disorders’ prevalence in Western countries, it is necessary to improve early detection and intervention to enhance developmental milestones. This systematic review identified the most effective screening instrument, which can be used at an early age and which identifies the maximum number of autism cases. We identified several instruments with adequate predictive properties—the Autism Parent Screen for Infants (APSI), Battelle Development Inventory, second edition (BDI-2); Brief Infant-Toddler Social and Emotional Assessment (BITSEA); First Year Inventory (FYI); Infant-Toddler Checklist/Communication and Symbolic Behavior Scales Developmental Profile (ITC/CSBS-DP); Program of Research and Studies on AUTISM (PREAUT-Grid); Checklist for Early Signs of Developmental Disorders (CESDD); Social Attention and Communication Study (SACS); and the Screening Tool for Autism in Toddlers and Young Children (STAT)—that can be applied from 12 months of age in Western countries. The ITC/CSBS-DP has been proposed for universal screening from 12 months of age onwards, complemented by the Modified Checklist for Autism in Toddlers, Revised/Revised with Follow-Up (M-CHAT-R/F), which can be used from 15 months of age onwards. This strategy could improve early detection in at-risk children within the current health system, thus allowing for early intervention.

## 1. Introduction

Autism spectrum disorders (ASDs) are neurodevelopmental pathologies that manifest as deficits in certain fundamental areas. The diagnostic requirements for ASDs have just two criteria, namely A) deficits in communication and social interaction and B) restricted and repetitive behaviors and interests, as shown by the current Diagnostic and Statistical Manual of Mental Disorders, Fifth Edition (DSM-5) diagnostic scheme.

Considering data from the last decades, several studies have been published to support the hypothesis of an increased number of ASD cases. Prevalence differs in some populational studies from North America, indicating 1 out of 68 children in 2012 [1] or 1 out of 59 in 2014 [2]. The most recent published study indicated that ASD prevalence was 13.4 per 1000 children aged 4 years in 2010, 15.3 in 2012, and 17.0 in 2014. It has been proposed that the average age of diagnosis is three or four years of age, or even later for children of low socioeconomic status or without a previous family history of ASD. The period between initial suspicion of ASD and final diagnosis can be a stressful and confusing time for families, and early identification and intervention can address this issue [3]. Nonetheless, perhaps the most crucial reason that justifies the need to detect the first symptoms is that it allows for intensive early intervention that leads to a better long-term prognosis [4]. In fact, in retrospective reports, most parents of children with ASD recall concerns regarding their child’s development after the first year of life [5].

Although screening for ASD is recommended at 18 months using M-CHAT, according to studies that have been conducted with high-risk siblings, sufficient evidence has been found to affirm that there are differential behavioral markers of ASD between 12 and 18 months of age [6]. Additionally, the M-CHAT instrument performs with a low-to-moderate accuracy in children with developmental concerns [7].

For effective screening, both specific screening tools (designed to identify children with risk signs of ASD) and wide-range screening tools can be used. With this second approach, children with any developmental difficulties will be detected in a first examination, and specific ASD tools will be used in the follow-up on this group. More accurate screening tools are essential for proper early detection. The best tools are those with higher values for sensitivity and positive predictive values (PPV). Sensitivity refers to the proportion of children correctly identified as “high-risk”, and PPV is defined as the proportion of children with positive screening who finally receive a diagnosis [7].

## 2. Material and Methods

The present systematic review arises from the formulation of the following question:

In the general population, which is the most efficient instrument to identify the maximum number of cases at risk of having Autism Spectrum Disorder and that can, therefore, be used at the youngest age possible?

To solve this question, a systematic bibliographic search was carried out between the 20 and 21 January 2021 through PsycINFO, PubMed, Virtual Health Library, and Cochrane Library databases. The search was performed using the combination of keywords: (“ASD” OR “autism”) AND (“specificity”) AND (“positive predictive value” OR “PPV”) AND (“identification” OR “screen*” OR “early screening” OR “early diagnosis” OR “early detection” OR “early identification”) AND (“tool” OR “instrument”); these terms may be found in the keywords, title, or summary of the article. The search was limited to the past 15 years and focused on humans, without limits on language or document type.

A table was created to record the studies, including the following fields:

Screening instrument;

Age of application;

Sample used;

Sensitivity and specificity;

Positive predictive value and negative predictive value.

## 3. Inclusion and Exclusion Criteria

We selected articles that referenced validated screening instruments, which were easily filled in by parents or daycare workers or applied by untrained observers, and articles that included measures of sensibility and specificity.

Discarded articles include those that did not discuss screening in young children or which did so through biological markers or evaluation software that requires a specific infrastructure.

These criteria were established because the goal of this study was searching for a low-cost, universal, early method of screening (preferably for children from 12 months of age). Low-cost refers to the fact that the screening instrument does not require a user license and a permanent economic expense for the public health service; also, that it is an easy and comprehensible tool that does not require prior specific training for the application by a clinical professional, and further, that the tool requires a short time for application and correction.

## 4. Data Extraction

In the database search carried out by a blinded and independent researcher, 357 records were found in PsycINFO, 323 in PubMed, 326 in the Virtual Health Library, and 1 result in the Cochrane Library that was discarded because it referred to the Fragile X Syndrome. There was a total of 1006 initial records, and after eliminating duplicates using the Mendeley reference program, 444 potentially eligible articles remained for the present review.

The selection process was carried out by two authors of this review, requiring, in case of doubt, the opinion of the third author. Preferred Reporting Items for Systematic Review and Meta-Analysis (PRISMA) guidelines were applied [8]. In the first selection phase, the title and summary of every article were selected by the established criteria. In this first phase, 248 articles were eliminated because the tools were not low-cost, leaving a total of 196 articles. All the screening tools that appear in these articles were analyzed, removing those that did not present sensitivity and specificity data. This process concluded with a selection of 34 articles that met all previously established criteria.

**Figure 1:**
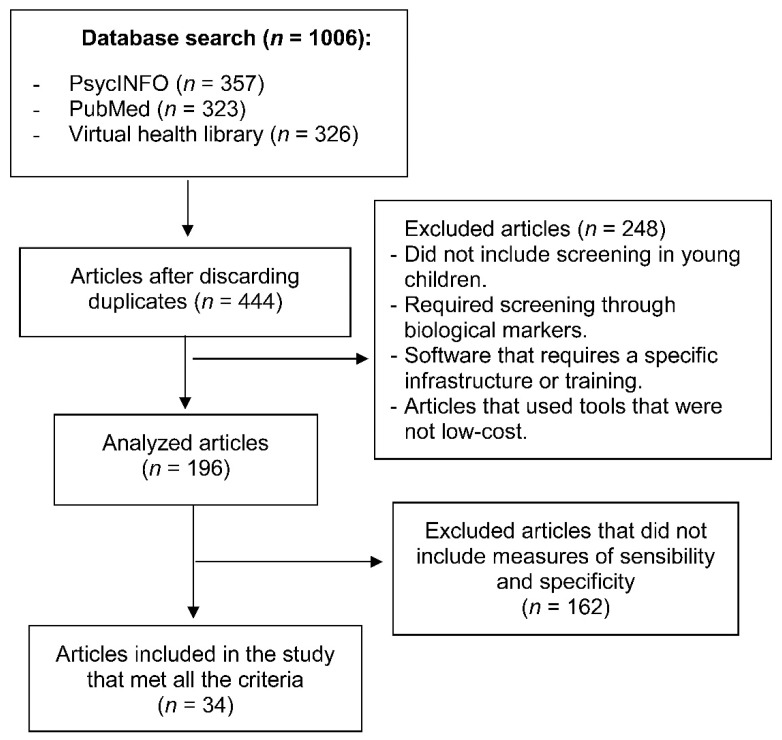
Schematic of the systematic review which includes the steps taken to collect all the studies included in the review.

The instruments found in the present review were compared with others proposed in previously published reviews [6,9], identifying screening tools that were not included in the present search and that had adequate predictive values. The search process is summarized in Figure 1.

## 5. Results

Table 1 shows the level 1 screening instruments applied from 12 months of age that met the abovementioned inclusion criteria. Screening instruments that can be applied before 18 months are shaded.

Among all the instruments identified in this work, the following instruments stand out for their predictive value and their easy and early application from 12 months of age:

BDI-II (Battelle development inventory, second edition): Evaluates children from 12 months to 8 years [12]. It helps measure a child’s progress along this developmental continuum by both global domains and discrete skill sets. The screening tool takes approximately 10–30 min to complete.

BITSEA (Brief Infant-Toddler Social and Emotional Assessment): Evaluates children from 11 to 48 months old. The 42-item questionnaire aims to identify children with social-emotional problems, behavior, or deficits in social and emotional competence [44]. The screening takes approximately 30 min to complete.

FYI: (First Year Inventory): This is a screening parent-report questionnaire [24]. It is a 63-item questionnaire developed as a general population-screening tool to identify 12-month-old infants who might be at risk for autism spectrum disorder (ASD) or a related developmental disorder. The screening takes approximately 30 min to complete.

ITC/CSBS-DP (Infant-Toddler Checklist/Communication and Symbolic Behavior Scales Developmental Profile): This is a 24-item questionnaire used as a screening tool for children from 9 months old and is a part of the evolutionary profile of the CSBS-DP scales. It was originally developed as a measure of language detection and evaluates social and communication behaviors [45]. It takes 10 min to complete.

PREAUT-Grid (Program of Research and Studies on AUTISM): A 10-item test to be administered by the pediatrician from observing how the child interacts with the pediatrician and his mother [37]. Evaluates children from 4 to 24 months old.

APSI (Autism Parent Screen for Infants): Evaluates children from 6 to 24 months old [10]. It is a 26-item forced-choice (yes, sometimes, no) parent-report questionnaire designed to monitor early signs of ASD in infants aged 6–24 months and takes approximately 10–15 min to complete.

CESDD (Checklist for Early Signs of Developmental Disorders): A 12-item checklist completed by nursery staff for children from 3 to 39 months [19]. It takes 10 min to complete.

SACS (Social Attention and Communication Study): A 15-item tool completed by clinicians to assess children from 8 to 24 months old [39]. It takes 10 min to complete.

STAT (Screening Tool for Autism in Toddlers and Young Children): A tool for children from 12 to 23 months old [40]. It consists of 12 activities assessing play, communication, and imitation skills and takes 15–20 min to administer by a trained clinician.

Table 2 includes the screening instruments found in this search that were valid to be used in children after 2 years.

In order to analyze the risk of bias in the eligible studies, an assessment was done, according to the Cochrane Handbook for Systematic Reviews of Interventions Version 5.1.0. Details are shown in Supplementary Table S1. These points need to be taken into account: (A) The samples included in the studies were largely heterogeneous from the general population, including children from different groups with ASD, OPD, or typical development. (B) Several studies were not using a control group. (C) There were limitations of sample size in several studies, which makes it hard to conclude the predictive ability of the screening instruments in larger populations. (D) Different patient ages in the groups were found that may affect the results. (E) Some studies indicated that the scale was performed by the parents alone and other studies by a child psychologist during a home visit or at the clinic.

## 6. Discussion

The primary goal of this review was to identify currently available tools for the early detection of ASD to provide the healthcare community with reliable and valid screening instruments that have a demonstrated efficacy for research and clinical practice in several populations. There is a pressing need to improve the early detection of autistic spectrum disorders, but we need to keep in mind that a very early detection means assuming a higher number of false positives, so the ethical implications of an early detection program must be taken into account. A false positive may cause unnecessary suffering for the family in question and an avoidable cost for the intervention to the community and health system. Notwithstanding, the considerable benefits of early intervention justify improving this strategy [54]. It is also advisable to have a good network of psycho-social support that helps the family to integrate the news of “suspicion of a child at risk for ASD”, thus avoiding an excessive negative emotional impact on the parents. Despite the limited research that exists in this area, an adequate intervention must be ensured for parents to maintain emotional adjustment and adaptation, even after diagnosis [55].

The initial symptoms of ASD become evident between 12 and 18 months of age, lasting throughout life, and a multifactorial etiology is involved, including familial predisposition and genetic risk factors, interacting with environmental factors that trigger or modulate the severity of the disorder [56]. Investigations have already suggested that early intervention from 6 months of age could lead to observable improvements in central areas of ASD, being a sufficient argument for checking all at-risk babies before being formally diagnosed at 30–36 months of age [57].

The present research attempts to identify the most efficient screening tool to better identify the cases at risk for ASD before 18 months of age, resulting in a thorough review of the scientific literature from the last 15 years. It is worth noting that not only predictive values have been taken into account to select the tool, but also applicability, validity, and the cultural environment where it will be used.

Among the different instruments used for the screening of early signs of autism in children from 12 months of age, the following instruments were selected due to their predictive value and their early application: the Autism Parent Screen for Infants (APSI), Battelle Development Inventory, second edition (BDI-II); Brief Infant-Toddler Social and Emotional Assessment (BITSEA); First Year Inventory (FYI); Infant-Toddler Checklist/Communication and Symbolic Behavior Scales Developmental Profile (ITC/CSBS-DP); Program of Research and Studies on AUTISM (PREAUT-Grid); Checklist for Early Signs of Developmental Disorders (CESDD); and the Screening Tool for Autism in Toddlers and Young Children (STAT). This research sought to reveal better detection instruments to identify children at risk of having ASD at an early age, improving the system used by the public health system. Currently, the detection system is primarily based on concerns from parents that become worried about their child’s development or behavior, and on a second step if the developmental delay is very evident to the health professional during the health checkup of every child, resulting in an important number of at-risk children presenting early markers of ASD who could be left undiagnosed during an essential period. In the universal screening protocols also used in several countries, children with ASD symptoms should be detected between 18 and 24 months, although some findings indicate that the average age of the diagnosis occurs much later, reducing the possibility of access to an early intervention program that is essential in the first months of life [58].

In order for a detection tool to be considered effective, it must not only demonstrate a strong set of psychometric properties but must also be easy to use in healthcare settings and easily integrated into everyday short procedures. Because of the limited resources available to pediatric healthcare professionals (for example, time per patient or available support staff), screening tools should be short, easy for parents to understand, and quick to correct by healthcare staff [59]. It will, therefore, be a difficult task to establish a screening program without also considering all the characteristics of the circumstances and context in which it will be implemented. The well-baby follow-up strategy, which was implemented following international practices and which aims to periodically follow up every child following birth to evaluate their health parameters, provides an exceptional framework to perform a universal screening of ASD in young babies [60].

Taking all this evidence into account, an instrument that can potentially improve the detection of ASD may be the ITC/CSBS-DP questionnaire, to be used as a screening tool within the routine 12-month well-baby follow-up program in Western countries. Even though it is possible to use for children from 6 months of age, the recommendation is to use it at the 12-month visit, where there are better predictive values for this tool [25,61]. The ITC/CSBS-DP tool is available in many languages, short, easy to apply, and can be applied by parents, medical doctors, nurses, or pediatricians. Since many children do not attend kindergarten, it would not be advisable to establish a general screening instrument such as the CESDD that is based on information supplied by kindergarten workers, since it would mean leaving out a considerable number of children from this screening of all those not attending kindergarten. The ITC/CSBS-DP is recommended above other tools such as the PREAUT Grid because of its ease of use when applied directly by parents and because it does not require an observational evaluation system that requires prior professional training. ITC/CSBS-DP is also recommended above the FYI or BISTSEA because, despite having good predictive data, the length of both tests (63 or 42 items) is much longer than the ITC/CSBS-DP, at 24 items, facilitating its addition to the routine well-baby follow-up visit. BDI-II was discarded, even though it has a very high sensitivity—but a very low specificity—though it could be an adequate tool for wide-range screening; however, the time required for its application makes it difficult to be chosen as a universal screening tool.

The ITC/CSBS-DP is a tool initially designed to evaluate language, attending to social and communication behaviors, but which does not address other specific factors of ASD such as repetitive behaviors, unusual sensory reactions, or other signs. Previous research has already supported that people with ASD have more difficulties in adaptive skills than other children with typical or atypical non-ASD development [62], so it is an adequate tool to detect this disorder. In detected at-risk cases, it is advisable to use another tool to improve follow-up of children at the second level, using tests that have been already validated to detect ASD within a large sample of children. To complete the detection and confirm ASD in children who have been detected during the screening, the M-CHAT tool is already integrated into health systems, but a better version is the follow-up interview called the M-CHAT-R/F, which is useful to detect ASD from 16 months of age [63]. The identification of at-risk children from the general population screening with the ITC/CSBS-DP test was described several times in previous studies and would be sufficient to initiate efficient early intervention to improve affected areas at an early developmental stage when intervention has demonstrated better results [61,64].

Special attention needs to be given regarding the sensitivity, specificity, PPV, and NPV of the different scales. The various indices of diagnostic accuracy of the different screening instruments are summarized in Table 1; Table 2 for two different age groups separately. The clinical significance of the various indices of diagnostic accuracy was evaluated by Cicchetti [65] and established as follows: <0.70 = poor; 0.70–0.79 = fair; 0.80–0.89 = good; 0.90–1.00 = excellent. Applying these criteria to the results in Table 1, not a single screening instrument, over the whole age range or for the younger and older subgroups, demonstrated good diagnostic accuracy for all four indices (Sensitivity-Se, Specificity-Sp, Negative predictive value-NPV, and positive predicted Value- PPV). These properties are extensively reported in the validation studies of the M-CHAT, M-CHAT R/F, ITC/DSBS-DP and CBCL 1.5-5. There is only one study including information on these properties for the other tools (see Table 1 for the specific values). Seven scales did not report any positive or negative predictive values. Moderate to high predictive values were reported on the scales for which the sensitivity, specificity, PPVs, and NPVs were indicated, although for those applied in an early screening from 12 months of age, the ITC/CSBS-DP results can be considered more stable compared to other measures. However, according to the results, many tools need further and deeper exploration of these properties.

Several screening tools required payment for use (e.g., the CARS-2, SCQ, SRS-2, STAT, ASQ-3, BDI-ST, BITSEA, and PEDS). Copyright laws prohibit their use without purchase, which prevents many researchers from using these standardized and validated tools. Moreover, only a licensed psychologist is permitted to purchase several of these tests. Furthermore, relatively substantial costs are often unaffordable for use in low-resource settings and more difficult for screening at the population level. A few exceptions are available to download freely, such as the AQ, APSI, CESDD, FYI, M-CHAT R/F, ITC, POSI, SSI, and SACS-R.

The tools included in this revision often consist of 10–118 items. The tools with fewer items to answer, such as the ITC/CSBC-DP, CESDD, PREAUT-Grid, SACS, and STAT, are more efficient than the others and require far less time to complete (5–10 min).

Taking into account the risk of bias assessment performed in the eligible studies, with the domains assessed being sequence generation (selection bias), allocation sequence concealment (selection bias), blinding of participants and personnel (performance bias), blinding of outcome assessment (detection bias), incomplete outcome data (attrition bias), selective outcome reporting (reporting bias), and other potential sources of bias, then the best instruments that scored 5 or 6 points, i.e., the highest scores, were these seven scales: the BDI-II, CBCL, ITC, M-CHAT, M-CHAT-F, M-CHAT-R/F, and TIDOS. In terms of validity, these screening methods have acceptable sensitivity rates, ranging from 70–100%, and specificity of between 80% and 100%.

M-CHAT and ITC/CSBC-DP are, within the low risk of bias tools, more accessible instruments, being freely available in different languages to download from the internet. From these validated tools, the Infant-Toddler Checklist (ITC/CSBS-DP) displayed a good consistency in early screening found in the bibliography and demonstrated a low risk of bias [25,61,64,66]. This 24-item tool is administered with good results in the general population from 12 months of age, which analyzes seven clusters: emotion and use of eye gaze, use of communication, use of gestures, use of sounds, use of words, use of objects, and understanding of words. The infants who score less than 10 points in any of the seven clusters are identified, and then followed up and subjected to further evaluations. The sensitivity and specificity of the ITC/CSBS-DP are reported as 78% and 84%, respectively [25]. Anyone can use it to predict ASD and non-ASD communicational difficulties in their toddlers quickly, cheaply, and easily.

## 7. Conclusions

In this study, we have identified the most effective screening tools to adequately detect ASD risk at 12 months of age. However, despite the bibliographic search carried out, some tools could have been left out of the study that could also be considered for the early identification of ASD, which could be the main limitation of this review. We consider that the proposal for early detection at 12 months with the ITC/CSBS-DP as a screening instrument is sufficiently justified by the high benefits for those affected by ASD and the low cost of implementing the screening in a health system setting, such as the well-baby care, which systematically evaluates all children at 12 months of age. We must also add that since there has been no validation of this scale across different populations, in addition to proposing the use of this instrument as a screening tool, we also propose the assessment of its validity and psychometric properties in different populations.

## Figures and Tables

**Table 1:** Screening instruments for a population under two years of age.

Instrument	Administration Age (Months)	Used Sample (Children)	Sensitivity	Specificity	Positive Predictive Value (PPV)	Negative Predictive Value (NPV)	Reference
APSI	6–24	204 high-risk, 79 low-risk controls	0.67	0.87	6–8 months: 0.43 21–24 months: 0.79	0.87–0.99	[10]
BeDevel	18–42	155 (75 ASD, 55 NT, 25 OPD)	0.83	0.81	0.80	0.83	[11]
BDI-II	0–95	604 ASD, 1064 NT	0.94	0.31	---	---	[12]
BISCUIT	17–37	1007 risk of ASD	0.93	0.86	…	…	[13]
BITSEA	11–48	223 ASD, 289 non-ASD	Up to 24 months: 0.91	Up to 24 months: 0.80	---	---	[14]
CASI (for Indi)	18-120	405 (75 ID, 83 ASD, 87 OPD, 160 NT)	0.89	0.89	0.67	0.96	[15]
CBCL 1.5-5	18–71	226 ASD, 163 OPD	0.74	0.53	---	---	[16]
CBCL 1.5-5 (Withdrawn PDP Scale)	18–59	101 ASD, 117 NT	Withdrawn: 0.89 PDP: 0.85	Withdrawn: 0.92 PDP: 0.90	Withdrawn: 0.90 PDP: 0.88	Withdrawn: 0.90 PDP: 0.87	[17]
	18–59	80 ASD, 103 OPD	Withdrawn: 0.88 PDP: 0.83	Withdrawn: 0.63 PDP: 0.60	Withdrawn: 0.65 PDP: 0.62	Withdrawn: 0.87 PDP: 0.82	[18]
CESDD	3–39	357 risk of ASD or language problems	0.92 and 0.90	0.73 and 0.68	0.19 and 0.32	0.99 and 0.98	[19]
CS-TSA	18–60	27 ASD, 41 OPD, 64 NT	Section 1: 0.89 Section 2: 0.78	Section 1: 0.68 Section 2: 0.79	---	---	[20]
DBC-ES	18–48	*n* = 142 ASD or PDD *n* = 65 OPD	0.83	0.48	0.78	0.56	[21]
Screening questionnaire developed for Taiwan	18–24	18 ASD, 59 NT	1	0.96	0.90	1	[22]
ECSA (Brief)	18–60	69 ASD	0.89	0.85	---	---	[23]
FYI	12	38 ASD, 15 NO-ASD, developmental delay 40 NT	0.92	0.78	0.74	0.93	[24]
ITC/CSBS-DP	6–24	5385 general population	0.93	0.89	12–24 months: +0.70	12–24 months: +0.87	[25]
M-CHAT	16–30	Sample 1: 2480 High- and low-risk Sample 2: 2055 low-risk	Both samples: 1	Both samples: 0.98	M1: 0.35 M2: 0.19	1	[26]
		122 risk of ASD, 106 NT	0.86	0.80	0.88	---	[27]
		966 born very premature	0.52	0.84	0.20	0.96	[28]
		109 language delay 732 NT	0.90	0.99	0.96	0.99	[29]
M-CHAT/F	16/30	341 positive for M-CHAT	0.55	0.79	0.78	---	[30]
M-CHAT-R/F	16–30	15,612 general population	0.96	0.86	0.47	0.99	[31]
M-CHAT-JV/F	16–30	1851 general population	0.47	0.98	0.45	---	[32]
PAAS	18–48	105 general population	0.88	0.93	0.95	0.84	[33]
PDQ-1	18–36	42 ASD, 38 OPD, 100 NT	0.85	0.99	0.88	0.99	[34]
POEMS	1–24	108 high-risk	0.74	0.73			[35]
POSI	16–48	232 children (16–36 months)	0.83	0.75	---	---	[36]
PREAUT Grid	4–24	4755 general population	P-4: 0.16 a 0.21 P-9: 0.30 a 0.41 P-24: 0.40–0.41	P-4: 0.99 P-9: 0.99 P-24: 0.99	P-4: 0.25 a 0.26 P-9: 0.20 a 0.36 P-24: 0.26 a 0.27	P-4: 0.99 P-9: 0.99 P-24: 0.99	[37]
Q-CHAT-10	18–24	126 ASD	0.91	0.89	0.58	---	[38]
SACS	12–24	20,770 general population	0.69 a 0.83	0.99	0.81	---	[39]
STAT	12–23	71 siblings with ASD	0.95	0.73	0.56	0.97	[40]
SORF	18–24	*n* = 84 ASD, *n* = 82 OPD, *n* = 62 NT	0.80	0.78	0.81	0.78	[41]
TIDOS	18–60	*n* = 86 ASD *n* = 76 OPD *n* = 97 general population	0.95	0.85	0.91	0.90	[42]
YACHT-18	18	2814 general population	0.60 ASD, 0.82 other developmental disorders	0.86 for developmental disorders	---	---	[43]

ASD: Autism Spectrum disorder. APSI: Autism Parent Screen for Infants. BDI-II: Battelle Developmental Inventory, second edition. BISCUIT: Baby and Infant Screen for Children with autism Traits. BITSEA: Brief Infant-Toddler Social and Emotional Assessment. CBCL: Child Behavior Checklist. PDP: Pervasive Developmental Problems. CESDD: Checklist for Early Signs of Developmental Disorders. CASI: Chandigarh Autism Screening Instrument. CS-TSA: “Chestionarul de Screening Pentru Tulburări de Spectru Autist”. DBC-ES: Developmental Behavior Checklist: Early Screen. ECSA: Brief Early Childhood Screening Assessment. FYI: First Year Inventory. ID: Intellectual Disability. ITC/CSBS-DP: Infant-Toddler Checklist/Communication and Symbolic Behavior Scales Developmental Profile. M-CHAT: Modified Checklist for Autism in Toddlers. M-CHAT-R: Modified Checklist for Autism in Toddlers, Revised. M-CHAT-R/F: Modified Checklist for Autism in Toddlers, Revised with Follow-Up. M-CHAT-JV/F: Japanese Version of the Modified Checklist for Autism in Toddlers, Revised with Follow-Up. NT: Normotypic. OPD: Other Pervasive Disorders. PAAS: Pictorial Autism Assessment Schedule. PDQ-1: Psychological Development Questionnaire-1. POEMS: Parent Observation of Early Markers Scale. POSI: Parent’s Observational Screen of Social Interactions. PPV: Positive predictive value. NPV: Negative predictive value. PREAUT Grid: “Programme de Recherches et d’Etudes sur l’Autisme”. P-4, P-9, P-24: PREAUT 4, 9 or 24 months. Q-CHAT-10: Quantitative Checklist for Autism in Toddlers. SACS: Social Attention and Communication Study. STAT: Screening Tool for Autism in Toddlers and Young Children. TIDOS: Three-Item Direct Observation Screen. YACHT-18: Young Autism and Other Developmental Disorders Checkup Tool. Shaded: Screening instruments that can be applied before 18 months.

**Table 2:** Screening instruments used in children older than 2 years.

Instrument	Administration Age (Years)	Sample Used	Sensitivity	Specificity	Positive Predictive Value (PPV)	Negative Predictive Value (NPV)	Reference
AQ-10 Child	4–11	432 ASD	0.95	0.97	0.94	---	[38]
ASRS	2–18	37 ASD, 30 OPD	Parents: 0.64 Teachers: 0.52	Parents: 63.30 Teachers: 0.71	Parents: 0.68 Teachers: 0.61	Parents: 0.59 Teachers: 0.62	[46]
BASC-2 (PRS-P)	2–5	224 positive ASD screening	0.76	0.73	0.86	0.57	[47]
CASD CASD SF	3–17	469 ASD, 138 OPD	CASD: 0.86 CASD SF: 0.95	CASD: 100 CASD SF: 0.96	…	…	[48]
CAST	4–12	1496 general population	Complete version: 0.83 Reduced version: 0.85	Complete version: 0.92 Reduced version: 0.91	Complete version: 0.63 Reduced version: 0.61	---	[49]
EDUTEA	3–12	2660 general population	0.87	0.91	0.86	0.99	[50]
M-CHAT and JA-OBS	2–3	3999 general population	0.90	---	0.96	---	[51]
OERA	3–10	76 ASD, 23 non-ASD	0.92	0.91	0.92	---	[52]
SCQ	3–7	219 premature children	0.91	0.86	0.31	0.99	[53]

AQ-10: Short Autism Spectrum Quotient. ASD: Autism spectrum disorder. ASRS: Autism Spectrum Rating Scales. BASC-2: Behavior Assessment System for Children, Second Edition. PRS-P Scale: Parent Rating Scales—Preschool. CASD: Checklist for Autism Spectrum Disorder. CASD-SF: Checklist for Autism Spectrum Disorder, Short Form. CAST: Childhood Autism Spectrum Test. M-CHAT: Modified Checklist for Autism in Toddlers. JA-OBS: Joint Attention Observation of Toddlers. OPD: Other Pervasive Disorders. OREA: Structured Observation for Autism Screening (acronym in Portuguese). PPV: Positive predictive value. NPV: Negative predictive value. SCQ: Social Communication Questionnaire.

## Data Availability

This study do not report any data.

## Supplementary Materials

The following are available online at https://www.mdpi.com/2227-9067/8/2/164/s1, Table S1: Risk of bias assessment.
