# Supplementary material for: Detection of Early Warning Signs in Autism Spectrum Disorders: A Systematic Review
Source: Children (Basel). 2021 Feb 22;8(2):164. doi: 10.3390/children8020164 (PMC7926898; doi:10.3390/children8020164)
Supplement: Supplementary file 1 [file children-08-00164-s001.pdf]

Supplementary Materials

Table S1. Description of domains measured to assess risk of bias. Cochrane Handbook for Systematic Reviews of Interventions Version 5.1.0. [1]

Domain	Description	High Risk of Bias.
1. Random sequence generation (selection Bias)	Method used to generate the allocation sequence is described in sufficient detail to allow an assessment of whether it should produce comparable groups.	Selection bias due to inadequate generation of a randomized sequence.
2. Allocation concealment (selection Bias)	Describes the method used to conceal the allocation sequence in sufficient detail to determine whether intervention allocations could have been foreseen in advance of, or during, enrollment.	Selection bias due to inadequate concealment of allocations prior to assignment.
3. Blinding of participants and personnel (performance bias)	Systematic differences between groups in the care that is provided, or in exposure to factors other than the interventions of interest. Describes all measures used, if any, to blind the study participants and personnel from knowledge of which intervention a participant received. Provides any information relating to whether the intended blinding was effective.	Performance bias due to knowledge of the allocated interventions by participants and personnel during the study.
4. Blinding of outcome assessment (detection bias)	Systematic differences between groups in how outcomes are determined. Describes all measures used, if any, to blind outcome assessors from knowledge of which intervention a participant received. Provides any information relating to whether the intended blinding was effective.	Detection bias due to knowledge of the allocated interventions by outcome assessors.
5. Incomplete outcome data (attrition bias)	Systematic differences between groups in withdrawals from a study. Describes the completeness of outcome data for each main outcome, including attrition and exclusions from the analysis. States whether attrition and exclusions were reported, the numbers in each intervention group (compared with total randomized participants), reasons for attrition/exclusions where reported.	Attrition bias due to amount, nature or handling of incomplete outcome data.
6. Selective Reporting (reporting bias)	Reporting of all analyses and outcomes described. Systematic differences between reported and unreported findings	Reporting bias due to selective outcome reporting.
7. Other Bias	Other threats to internal validity. Any important concerns regarding bias not addressed above. If particular questions/entries were pre-specified in the study's protocol, responses should be provided for each question/entry.	Bias due to problems not covered elsewhere in the table.

Reference:

1. Deeks, J. J., Higgins, J. P. T., Altman, D. G., and Green, S. (2011). Cochrane Handbook for Systematic Reviews of Interventions Version 5.1.0. Available at: http://training.cochrane.org/handbook
